# Signaling effect in social network and charity crowdfunding: Empirical analysis of charity crowdfunding of Sina MicroBlog in China

**DOI:** 10.3389/fpsyg.2022.944043

**Published:** 2022-10-13

**Authors:** Chaoyang Li, Xinyi Li, Jingmei Wang, Mengyang Pan, Weiyi Gao

**Affiliations:** School of Management, Henan University of Technology, Zhengzhou, China

**Keywords:** online charity crowdfunding, crowdfunding performance, social capital, social recommendation, Sina MicroBlog, prosocial behavior, reciprocity

## Abstract

With the increasing number of online charity donations, research on the influencing factors of individual donation behavior has become an important topic. Social interaction information in crowdfunding has become an essential basis for potential backers to make decisions. It provides new research space for charity crowdfunding and social capital theory. The primary purpose of this study is to explore the influence of social capital, social recommendation, and other signals on charity crowdfunding performance. We obtain 4,780 project information on the charity crowdfunding of Sina MicroBlog through data collection procedures. Our research found that both external social capital and internal capital can significantly improve the fundraising performance of crowdfunding projects. Projects with more social recommendations are more likely to obtain financial support. In the case of Medical aid crowdfunding projects, the positive promotion effect of social recommendations on project fundraising ability is enhanced. To get more effective support for crowdfunding projects, it is necessary to pay attention to the construction of social capital and the cultivation of its reputation to obtain the recognition of potential backers.

## Introduction

Online crowdfunding expands the financing target to the general population, which provides new financing channels for innovation, enterprise production and operation, social welfare, and other activities. Especially in the current context of the COVID-19 pandemic, online charity crowdfunding, one of the forms of online crowdfunding, plays a vital role in charities. The 2020 China Charity Donation Report conducted by China Charity Alliance shows that Charity organizations in China raised more than 8.2 billion yuan through 20 online fundraising platforms in 2020. More than 10 billion people participated in online donations, highlighting the vitality of “Internet + Charity.” Small donations with civic participation have become a trend of charity contributions. Therefore, it is significant to study the influencing factors of individual donation behavior in the context of the Internet.

Charity Crowdfunding of Sina MicroBlog is one of largest online charity platforms in china which involve many charity crowdfunding projects such as medical needs, education assistance, and environmental protection. According to the report of *China Philanthropy Times* in 2021, over 7 million people donated on this platform and the funding were up to more than 700 million yuan. There is an abundance of data available in the charity crowdfund platform, including detailed information of charity crowdfunding projects, such as the target fund-raising amount and fund-raising time, and social interaction information, such as the fundraising experience of project sponsor and the forwarding and recommendation of project, which provide sufficient data for our research.

Current researches mainly focus on reward crowdfunding, and very little literature emphasis on charity crowdfunding (Zhang and Chen, 2019). Unlike reward crowdfunding, donors are not rewarded with money, and their funding motives are different. Existing literatures of charity crowdfunding study on emotions, values of donors and prosocial behavior, less playing emphasis on how the effect of social capital and social recommendation on the contributions of charity crowdfunding. Although some studies have paid attention to the impact of social capital on crowdfunding, it is still not clear enough to divide the boundary of social capital. These studies mainly discussed individuals’ willingness to charity crowdfunding from individuals’ external motivation and internal motivation. For example, Mollick (2014), Zheng et al. (2014); Colombo et al. (2015), Skirnevskiy et al. (2017) and other studies focus on reward crowdfunding projects, and do not divide social capital into external and internal dimensions. As another example, Bagheri et al. (2019) also did not discuss the social capital variable. Their work suggests that information sharing, values, ideas and beliefs, learning ability, and other factors affected the number of individual donations in charity crowdfunding (Bagheri et al., 2019).

Meanwhile, previous studies have focus on the historical information of the project sponsor, and lack for taking the dynamic sharing information of the current crowdfunding project into consideration (Skirnevskiy et al., 2017). It is essential to consider social recommendations of the crowdfunding project. Peng et al. (2022) found that the number of subsequent participants’ donations was influenced by the emotions of text messages on the crowdfunding platform. The contributions of real-name donors are higher than anonymous donors. However, this study does not involve project recommendation information. Sura et al. (2017) and Li et al. (2018) discussed the impact of project characteristics and platforms on project fundraising capabilities with questionnaires. Their researches are relatively ignoring social attributes of projects. Current researches ignore individual characteristics and information such as the social connection between users.

Moreover, most researches are based on crowdfunding platforms of United States or Europe. Cultural differences between Chinese and European, and American make the previous research not applicable to charity crowdfunding in the context of China (Zheng et al., 2014).

Our study intends to filling those gaps. We collect data on Charity Crowdfunding projects of Sina MicroBlog through a data crawler program and divides social capital into internal and external. Based on the above work, we examine the influence of sponsors’ social capital and potential backers’ social recommendation on project fundraising performance. Firstly, we define the types of social capital, and consider the impact of external and internal social capital on the performances of charity crowdfunding. The participant type of crowdfunding projects can be divided into social interaction information between potential backers and project sponsors and social interaction information between potential backers and projects. Social interaction information between potential backers and project sponsors can be measured by social capital (Cai et al., 2021). Our research subdivides social capital into two dimensions: external social capital and internal social capital. Secondly, we consider the impact of social recommendation on crowdfunding performance. Potential backers can forward and recommend their preferred projects, reflecting users’ social recommendation behavior (Kuppuswamy and Bayus, 2017; Madrazo-Lemarroy et al., 2019). Finally, we also take the effect of different project types on crowdfunding performance into account.

The remainder of this paper is arranged as follows: The second section describes theoretical model analysis; The third section introduces the literature and hypotheses; The fourth section is the study design; The fifth section discusses reports results; Finally, we provide our conclusions and implications.

## Theory and model analysis

The information asymmetry in online charity crowdfunding is an important factor limiting donation activities, which lead to charity crowdfunding projects inefficient (Donovan, 2021). It is more complex to execute formal contracts in online charity crowdfunding than in traditional trading. While, the social information and historical transaction behavior of project sponsor can be observed by potential backers before online crowdfunding transaction. On this basis, potential backers can make a fundamental judgment on project sponsor’s credit. Therefore, the opportunistic of Information dominant party will be effectively restrained, which can facilitate resource access to crowdfunding projects (Hildebrand et al., 2017). The informal institution formed by interactive communications between donors and beneficiary can effectively fill the lack of governance in the formal system (Lins et al., 2017). Hence, we can investigate influencing factors of charity crowdfunding based on interactive information of crowdfunding platform.

The lack of constraints on the opportunistic behavior of participants will lead to the loss of overall social welfare in online crowdfunding. Signaling from social networks is vital to relieve this prisoner’s dilemma. Social capital is endogenous because of relational structures in social networks. Resource functions and institutional effects, which are important attributes of social capital, play a vital role in governing modern social organizations and making decisions by donors in the network environment (Peng et al., 2022). Social recommendations reflect user’s approval of a product or service, which is a social resource generated from informal relationships. The higher the social recommendations are, the more likely market accepted the product (Kromidha and Robson, 2016). In a crowdfunding campaign, social recommendations are mainly reflected by the “likes,” “reposts” and other signals of social friends (Liu et al., 2018). The higher social recommendations are, the more likely the crowdfunding project will be accepted.

Participants can make an appropriate decision based on the social network information and the historical data of the opponent. If one party often adopts an opportunistic strategy, it will lose the opportunity to cooperate in the future. As a result, total social welfare will be reduced. Based on this consideration, it improves the classic Prisoner’s Dilemma model with a social network-based game model. The relationship connection strength in the social network is λ, which can reflect the level of social capital. The network signal disclosure quality is θ, which describe the degree of social recommendation. The game structure is given in Table 1.

**TABLE 1:** Payment matrix of two parties in social network.

		Player 2	
		
		Cooperative	Non-cooperative
Player1	Cooperative	$\frac{1}{1-\delta }\,c$,$\frac{1}{1-\delta }\,c$	*b*,$a+\frac{\delta \,\left[1-p\,\left(\lambda ,\theta \right)\right]\,c}{1-\delta }$
	Non-cooperative	$a+\frac{\delta \,\left[1-p\,\left(\lambda ,\theta \right)\right]\,c}{1-\delta }$,*b*	0,0

In the classic Prisoner’s dilemma, the game result is (*c*,*c*) when both parties adopt the cooperative strategy, which means both parties gain *c*. The game result is (*a*,*b*) when one party cooperates and one party takes opportunistic behavior. Under these conditions, the cooperative party gains *b*, while the betraying party gains *a*. The payoffs satisfy the conditional constraints in the prisoner’s dilemma: *b* < 0 < *c* < *a*, and *a* + *b* < 2*c*. Because of individual rationality, betrayal is the equilibrium strategy of the game, and the social welfare maximization strategy of (cooperation, cooperation) will not appear.

We define ω = *c*−*a* as the project implementation level. When the conditions of the prisoner’s dilemma isω = *c*−*a* < 0, the parties have the possibility of betrayal. When ω > 0, the trade will be succeeded. When two parties play a multi-stage game, the discount rate of the parties is assumed to be δ(0 < δ < 1). In the current and subsequent future trade, the parties will maintain the cooperation strategy when “the return of implementing betrayal strategy” is less than “the sum of the discounted return of implementing cooperative strategies,” which is shown as follows:


(1) $$c+\delta \,c+\delta^{2}\,c+\delta^{3}\,c+\cdots >a$$

(2) $$S\,i\,n\,c\,e\,0<\delta <1,c+\delta \,c+\delta^{2}\,c+\delta^{3}\,c+\cdots =\frac{c}{1-\delta }$$

The parties will adopt a cooperative strategy under conditions of $a<\frac{c}{1-\delta }$ and the game result of cooperation strategy is ($\frac{1}{1-\delta }\,c$,$\frac{1}{1-\delta }\,c$).

Now we analyze the decision behavior of the parties in the social networking. The stronger ties in the social network, the more likely the sponsor’s betrayal strategy is to be found. In other words, the stronger the network signal sends, the more likely the sponsor’s betrayal strategy will be discovered. There is a certain probability that a betrayal will be detected. $\frac{\partial p\,\left(\lambda ,\theta \right)}{\partial \lambda }>0$, $\frac{\partial p\,\left(\lambda ,\theta \right)}{\partial \theta }>0$. The discovery probability *p*(λ,θ) is positively correlated with λ and θ. λ is the strength of relationship connection in the social network. θ is signal display quality of network. 1−*p*(λ,θ) is the probability that the party adopts a betrayal strategy without being discovered and makes the game decision in the next period.

If the party takes betrayal strategy in the first phase, they will keep the cooperation strategy from the second phase. On this condition, the return of the first phase is still *a*. The discounted return of the cooperation after the second phase is as follows:


(3) $$\delta \,c+\delta^{2}\,c+\delta^{3}\,c+\cdots =\frac{\delta }{1-\delta }\,c$$

If the betrayal strategy is not discovered, the expected return is the sum of the first phrase return and the later discounted return:


(4) $$a+\left[1-p\,\left(\lambda ,\theta \right)\right]•\left(\delta \,c+\delta^{2}\,c+\delta^{3}\,c+\cdots \right)$$

$$=a+\frac{\delta }{1-\delta }\,\left[1-p\,\left(\lambda ,\theta \right)\right]•c=a+\frac{\delta \,\left[1-p\,\left(\lambda ,\theta \right)\right]\,c}{1-\delta }$$

In this case, the return of the betrayer is $a+\frac{\delta \,\left[1-p\,\left(\lambda ,\theta \right)\right]\,c}{1-\delta }$ and the return of the partner is *b*. The result of the game is($a+\frac{\delta \,\left[1-p\,\left(\lambda ,\theta \right)\right]\,c}{1-\delta }$, *b*).

If the trade is guaranteed, the benefits of cooperation between the two parties are more significant than the benefits of non-cooperation, which is shown as follows:


(5) $$\frac{c}{1-\delta }>a+\frac{\delta \,\left[1-p\,\left(\lambda ,\theta \right)\right]\,c}{1-\delta }$$

(6) $$\mathrm{The}\,\mathrm{equivalent}\,\mathrm{inequality}\,\mathrm{is}\,\frac{1-\delta \,\left[1-p\,\left(\lambda ,\theta \right)\right]}{1-\delta }\,\text{c-a}>0$$

(7) $$\mathrm{That}\,\mathrm{is}\,c-a>-\frac{\delta \,p\,\left(\lambda ,\theta \right)}{1-\delta }\,c$$

Since ω = *c*−*a* is the project implementation level, it requires $c-a=\omega >0>-\frac{\delta \,p\,\left(\lambda ,\theta \right)}{1-\delta }\,c$. The deal is made requires ω > 0. Due to the existence of social connections, the constraint condition ω of the trade is relaxed to $-\frac{\delta \,p\,\left(\lambda ,\theta \right)}{1-\delta }\,c$. The signaling effect enables some trade to be realized, which cannot been carried out previously.


(8) $$A\,c\,c\,o\,r\,d\,i\,n\,g\,t\,o\,i\,n\,e\,q\,u\,a\,l\,i\,t\,y\,\left(6\right),l\,e\,t\,U=c-a+\frac{\delta \,p\,\left(\theta ,\lambda \right)}{1-\delta }\,c$$

(9) $$T\,h\,e\,n\,\frac{\partial U}{\partial \lambda }=\frac{\delta \,c}{1-\delta }\,\frac{\partial p\,\left(\lambda ,\theta \right)}{\partial \lambda }>0$$

(10) $$\frac{\partial U}{\partial \theta }=\frac{\delta \,c}{1-\delta }\,\frac{\partial p\,\left(\lambda ,\theta \right)}{\partial \theta }>0$$

The two inequalities (9) and (10) indicate that the constraining force of trade will increase with the enhancement of the social relationships of project sponsors and the signal display level. The intensity of social connection and quality of network signal display will improve trade efficiencies. In online charity crowdfunding, we can infer that social capital can significantly improve the fund-raising ability of projects, and social recommendations can also promote the funding of crowdfunding projects. Next, we will analyze these two inferences.

## Literature review and research hypothesis

### Social capital signals and fundraising

Due to differences between social networks, their social capital is also unique. External social capital refers to social connections generated by social media associated with crowdfunding platforms, which are outside the trading platform and can be measured by the number of fans on related social platforms, such as the number of social friends on Facebook, LinkedIn, and other social media accounts. Internal social capital is interactions between potential backers in the trading platform, measured by the number of backers, endorsements, and projects supported on the crowdfunding platform (Kuppuswamy and Bayus, 2017; Madrazo-Lemarroy et al., 2019).

The inside or outside interactive information on Charity Crowdfunding on Sina Micro Blog provides an important informal institutional guarantee for crowdfunding campaigns. Outside interactive information of project sponsors, such as fans and numbers of views, can be found on Charity Crowdfunding of Sina MicroBlog, which displays social connections of project sponsor and can effectively increase the possibility of the project sponsor communicating with outside. The project sponsors inside interactive information, such as previously contributed money to crowdfunding projects, the number of backers they brought in, etc., can also be displayed on the crowdfunding platform. At this point, social capital is a crucial symbol of the project sponsor’s credit and quality (Donovan, 2021). High social capital reflects the better performance of project sponsors in past trading activities, representing personal credit and ability, and offering an important basis for decisions for potential backers (Zheng et al., 2014).

In terms of external capital, each social connection represents not only a project propagandist and potential backer but also a project supervisor (Calic and Mosakowski, 2016). When there is a high level of external social capital, the project sponsor will have more channels to connect with external platforms, and the more potential backers there are. Moreover, the project sponsor is subject to more supervision and is less likely to adopt opportunistic behavior.

Internal social capital mainly obtains resources based on the reciprocal relationship between the person who has received the support of the project sponsors and the project sponsor (Colombo et al., 2015). Receivers responsible and obligated to support the project sponsors’ project, which is called direct reciprocity in the reciprocity theory (Khadjavi, 2017). The integrity behaviors of the project sponsors in history will also gain the trust of individuals who are not direct beneficiaries. According to the reciprocity theory, indirect beneficiaries will also support project sponsors with the influence of indirect reciprocity. Consequently, the project will be widely spread, and obtaining funds will be more accessible (Colombo et al., 2015).

Whether external or internal social capital, social capital based on informal values or social norms recognized by groups can guide group members to cooperate and reduce transaction costs. Social capital also can restrain non-trustworthy behavior through external punishment mechanisms formed by public opinion in the social network (Mollick, 2014). The informal institutional constraints formed by social capital may be more critical than the formal system, especial when the formal system is weak or the contract execution cost is too high (Lins et al., 2017). Social capital can significantly improve the fundraising capacity of projects in online crowdfunding. Therefore, the following research hypothesis is proposed:

H1: Social capital has a positive effect on project fundraising ability.

H1a: External social capital has a positive effect on project fundraising ability.

H1b: Internal social capital positively affects project fundraising ability.

### Social recommendation and fundraising ability

Unlike traditional information dissemination, social networks mainly spread information through interactions including the emotions of participants of the project (Schafer et al., 2018). The signals of social recommendation contain more identity and trust to project sponsors, such as “likes” and “reposts” on social networks. Social recommendation promotes information aggregation and resource acquisition (Hong et al., 2018). A project will gain more understanding and commendation from social recommendations in an online crowdfunding campaign when it gets more “likes.” It can be relatively easy to obtain financial support from social recommendations (Wang et al., 2018). The social recommendation indicates a superior reputation, which illustrates a good market performance of this product. In this sense, it is an important basis for people to make decisions under information asymmetry.

During an online crowdfunding campaign, project sponsors with more social recommendations will get financial support more accessible. This incentive effect can reduce fundraisers’ short-term opportunistic behavior. In addition, decision-makers tend to ignore their private information and imitate others’ behaviors under information asymmetry (Banerjee, 1992). The phenomenon of herd behavior also exists in online crowdfunding. Potential backers are the most likely to follow others’ decisions to invest in the project with high social support under the effect of herd behavior. In this sense, a project with more social recommendations is relatively easy to obtain financial support earlier (Colombo et al., 2015).

Users can forward the project information to social platforms when they see a supported project on the Charity Crowdfunding of Sina MicroBlog. The fundraising channel could be expanded when more people know about this project. People from external social media can also learn about the progress and usage of fundraising. The social recommendation of the project enables the public to play a supervisory role in a crowdfunding campaign. The platform and project sponsors will improve information transparency to gain project credibility. Due to the signals of social recommendation, the risk perception of potential backers will be reduced, and the trustworthiness of the project will enhance (Skirnevskiy et al., 2017). Social recommendations will also spread project information widely, and more potential backers are aware of the project’s existence. In brief, the social recommendation can improve the fundraising ability of the project.

H2: Social recommendation has a positive effect on project fundraising ability.

### Moderating effect of project type on social recommendation signals and fundraising ability

Due to the context-dependence of individual decision-making, there may be some differences in their perception of identity brought by social recommendations (Li et al., 2021). The role of social recommendations on fundraising ability may differ when potential investors face different projects (Proelss et al., 2020). According to Maslow’s Hierarchy of Needs theory, physiological, and safety needs are people’s basic needs. In medical aid projects, it will threaten recipients’ life safety if they can’t get financial support in time. Consequently, people prefer to support these urgent and basic physiological needs projects (Proelss et al., 2020). Medical aid crowdfunding projects are often closely related to people’s basic needs, which will be preferred to share, spread, and identify with the public (Hong et al., 2018). Compared with non-medical aid projects, people have a more profound recognition and concern to interactions of medical aid programs. Therefore, medical aid projects are more likely to be funded.

Crowdfunding projects mainly include medical aid, environmental protection, and education assistance on the Charity Crowdfunding of Sina MicroBlog. The disease will bring a heavy blow to the patient’s physical and mental health and even threaten their life. Medical aid crowdfunding projects shared by the platform are more likely to be supported by users because of the urgent demand for funds. According to the previous analysis, projects receiving more social recommendations are more likely to get financial support. In the case of medical aid crowdfunding projects, the promotion effect of social recommendation on the project’s fundraising ability will be more obvious. Hence, Medical aid crowdfunding projects have a positive moderating effect between social recommendation and the project’s fundraising ability. The following research hypothesis is proposed:

H3: Project type plays a moderating effect on the relationship between social recommendation and project fundraising ability. In the case of medical aid crowdfunding projects, the promotion effect of social recommendation on project fundraising ability is enhanced.

## Materials and methods

### Data

Charity Crowdfunding of Sina MicroBlog is an online crowdfunding platform established earliest in China. The projects on the platform are authentic and reliable, benefiting from the standard procedures and strict review processes. This Crowdfunding platform contributes detailed and objective data to this research. Project information on this crowdfunding platform is mainly divided into three types: project information, social information of sponsor, and historical information of project sponsor. Project information includes the times of project forwarding, fundraising target amount explicitly, duration, project type, finally raised funds, and project fundraising ratio; The social information of the project sponsor involves the number of social friends and the number of views; Historical information of project sponsor refers to the number of projects they have supported, the number of backers they brought in, and charity points.

Since project sponsors’ social information and performances will vary over time, we need to consider the project information validity carefully. Then, this study selected projects during the time from January 1, 2016 to December 8, 2017. Project information mainly includes the project fundraising goal, the duration of project funding, the number of project funds obtained, the number of social friends, the number of “likes” and other information. In data processing, we removed 527 projects which have deactivated social accounts and tested informal projects. The projects with incomplete details are also removed. This research finally obtained 4,780 projects.

### Measurements

The dependent variable is the crowdfunding performance of the project, which is measured by the number of funds finally raised for the project, and represented by *Funding Raised* (Mollick, 2014). For the robustness test, this study also takes the proportion of funds raised for the project as the dependent variable (Lin et al., 2013). The proportion is the ratio of actual funds to target funds, defined as the *Funding Level*.

According to the research of Butticè et al. (2017), Hervé et al. (2019) and Madrazo-Lemarroy et al. (2019), the external social capital is measured by the number of Weibo page views. This indicator can reflect the number of a blogger’s active followers and the number of real connected users, indicating the blogger’s social capital outside of the crowdfunding platform. There are many inactive followers or fake followers on Weibo. These followers will not read, click a like, or comment after following Weibo bloggers, which cannot represent the external resources for bloggers. In this sense, we do not use the number of Weibo followers as the indicator of external social capital. Therefore, we select the number of Weibo views to measure external social capital. This is essentially consistent with the standard of external social capital by the number of social friends of project sponsors (Colombo et al., 2015). Referring to the research of Kim et al. (2017), Davies and Giovannetti (2018), and Madrazo-Lemarroy et al. (2019), the internal social capital is measured by two indicators. One of the indicators is the number of donors in previous projects of the sponsor; another is the charity points. The number of backers brought by sponsors is not just a measure of their fundraising ability but also a label of their trustworthiness. The charity points are based on project initiators’ participation in charity campaigns, which accumulated by Sina MicroBlog Charity Crowdfunding rules. Points can be gained from original or reposted topic words of Weibo charity projects and participation in numerous charity activities. The charity points can represent their social capital, which objectively reflects sponsors’ involvement in charity projects. The second indicator of internal social capital is consistent with the first one. The two indicators all represent the relationship between backers and sponsors. They can use to measure internal social capital (Colombo et al., 2015; Skirnevskiy et al., 2017).

Backers can forward crowdfunding projects on social media through Charity Crowdfunding of Sina MicroBlog. The social recommendation can be measured by the forwarding times of crowdfunding projects, which are interactions between backers and projects (Kromidha and Robson, 2016; Schafer et al., 2018). Potential backers can also recommend projects through the sharing mechanism to express their support for projects. This interaction signal between potential backers and the project can increase project trustworthiness. The more times a project is recommended, the more backers will be attracted.

The moderator variable is the project type, which is represented by *Type*. When the value of *Type* is 1, it means medical aid projects, and 0 represents other projects. In addition to the above-mentioned explanatory variables, many factors can affect fundraising ability. Based on similar literature, the control variables selected include crowdfunding goal, project duration, year of project implementation, and other indicators (Mollick, 2014). The crowdfunding goal is the amount of capital demand set by the project sponsor, which is represented by the *Goal*; The project duration is the days between the start and ends the of project, which is defined as Dur; To control the possible impact of the time factor on the fundraising ability, the year of the project is also controlled, represented by *Year*. When the value of *Year* is 1, it means 2017, and 0 represents 2016.

## Statistical analyses

### Descriptive analyses

The main continuous variables are winsorized at 1 and 99% to eliminate the influence of extreme values. A summary of specific variables is shown in Table 2. The mean value of the fundraising ability is 3,346.362, and the variance is 7666.479; the mean value of the fundraising completion ratio is 16.135%, and the variance is 30.474%. These two indicators show a big difference in the final amount of funds obtained by the project, and the overall completion level of the project is not high. The mean value of external social capital is 748,408.500, which indicates that some project sponsors’ Weibo pages have a higher number of views and are widely connected to the outside world. In contrast, others have less connection to the outside of the platform. As to the internal social capital, the standard deviation of charity points and the number of donations brought by project sponsors are enormous. The internal social capital of different project sponsors differs sharply. The mean of social recommendations is 42.455. On average, forwarding and sharing of projects are relatively active.

**TABLE 2:** Definition of main variables and descriptive statistics.

Variables	Definition	Mean	Std. dev.	Min	Max
*Funding raised*	The number of funds finally raised for the project	3346.362	7666.479	0	50254.000
*Funding level*	The percentage of a project’s funding level that is raised by founders	16.135%	30.474%	0	126.300%
*Goal*	The amount sponsors seek to raise	40418.540	32850.000	700.000	100000.000
*Duration*	The number of days between the start and the end of the project	54.516	13.113	0	60
*Year*	Dummy variable which equals the value 1 if the year is 2017, and 0 is 2016	0.335	0.472	0	1
*Type*	Dummy variable which equals the value 1 if the project type is medical aid, and 0 otherwise	0.839	0.367	0	1
*Fans*	The number of followers on the Weibo social platform	55630.100	161110.700	75	1274264
*Prefunding*	The number of projects which sponsors have created on the platform	2.5040	2.6404	0	9
*Extcapital*	The number of Weibo readings	748408.500	1096679	0	2560000
*Intcapital I*	The charity points	519296	5621687	6	1.55e + 07
*Intcapital II*	The number of backers brought by sponsors	7636.325	8891.148	0	20000
*Recommend*	The forwarding times of crowdfunding projects	42.455	65.601	2	474

### Regression analyses

To analyze the relationship between social connection, social recommendations, and other factors on fundraising ability, this study mainly adopts the following econometric model for regression analysis:


(11) $$\begin{array}{l} Funding\;Raised={\text{α}}_{0}+{\text{α}}_{1}Control+{\text{α}}_{2}Extcapital\\ \;\;\;\;\;\;\;\;\;\;+{\text{α}}_{3}Intcapital_{i}+{\text{α}}_{4}Recommend+\text{ε} \end{array}$$

*Control* refers to a group of control variables, including crowdfunding goal, duration, and other variables.*Intcapital*_*i*_ represents the two internal social capital, respectively: the number of Weibo views and the number of backers brought by sponsors. The variable description is shown in Table 2. To avoid bias from significant differences in variables and make the data more stable, we take the logarithm of these variables, such as crowdfunding goal, the number of followers, fundraising ability, external social capital, internal social capital, and social recommendation. We also applied the VIF test and found that the variance inflation factors were lower than 10, indicating no severe multicollinearity problem in this study.

First, we discuss the relationship between social capital and fundraising ability. The regression results are presented in Table 3. Model 1 is the regression result of the control variables, and Model 2 is the regression result of the explanatory variables and explained variables without adding control variables. Model 3 is the regression result of the explanatory variable and the explained variables with control variables. Even though other variables have been controlled, the regression results show that the coefficient between *Extcapital* and *Funding raised* is 0.184, *p* < 0.001. There is a significant positive correlation between external social capital and the fundraising ability of the project, and Hypothesis H1a is supported. The above results indicate that when the project sponsor has social connections outside the crowdfunding platform, the acquisition of funds for the project will be promoted. External social capital is a critical way to help a project obtain resources.

**TABLE 3:** Regression results.

	(1)	(2)	(3)	(4)
	Funding raised	Funding raised	Funding raised	Funding raised
*Goal*	−0.005 (-0.14)		0.009 (0.30)	0.017 (0.53)
*Dur*	0.006* (2.50)		−0.004 (−1.68)	−0.004 (−1.58)
*Year*	0.162* (2.48)		−0.0204 (−0.33)	−0.0214 (−0.35)
*Type*	0.298*** (3.29)		0.583*** (6.82)	0.507*** (5.65)
*Fans*	0.130*** (7.91)		−0.236*** (−10.26)	−0.233*** (−10.13)
*Prefunding*	0.0748 (1.53)		−0.462*** (−8.15)	−0.480*** (−8.41)
*Extcapital*		0.0325 (1.47)	0.184*** (7.01)	0.189*** (7.20)
*Intcapital I*		0.113*** (10.21)	0.111*** (8.61)	0.109*** (8.49)
*Intcapital II*		0.0477 (1.50)	0.116*** (3.37)	0.114*** (3.33)
*Recommend*		0.713*** (24.29)	0.789*** (26.53)	0.793*** (26.66)
Recommend *type				0.206^**^ (2.72)
*Constant*	4.763*** (14.47)	2.194*** (15.29)	2.161*** (6.74)	2.100*** (6.54)
Adj *R*^2^	0.034	0.174	0.204	0.205

*, **, ***Denote statistical significance at the 10, 5, and 1% levels, respectively. *T*-value is reported in parentheses.

The coefficient between *Intcapital I* and *Funding raised* is 0.111, *p* < 0.001. The coefficient between *Intcapital II* and *Funding raised* is 0.116, *p* < 0.001. These two regression results indicate a significant positive correlation between internal social capital and fundraising ability. Internal social capital can significantly promote crowdfunding projects to get funds. Hypothesis H1b is supported. The results also reveal that the social network inside the crowdfunding platform can provide valuable information for platform users. Internal social capital is an important signal of the trustworthiness of project sponsors and an important basis for potential backers to make a decision.

The results of model 3 show that the coefficient between *recommend* and *Funding raised* is 0.789, *p* < 0.001. There is a significant positive correlation between social recommendation and the fundraising ability of the project. Crowdfunding projects can be benefited from social recommendations significantly. Hypothesis H2 is supported.

**FIGURE 1:**
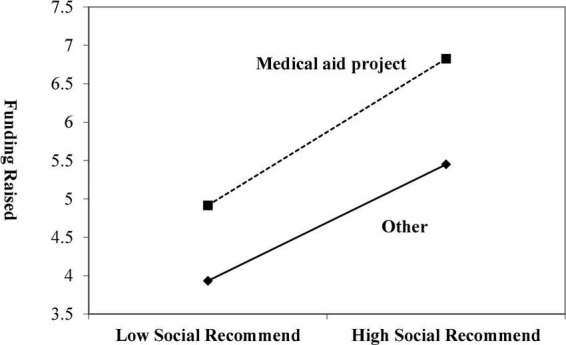
Moderating effects of project type.

Considering the context-dependence of potential backers’ decision-making, we introduce the project type as moderating variable. Then, we will explore the relationship between social recommendation and fundraising ability. We use *recommendation* and *Type* to make an intersection and put the intersection into the regression equation. The regression results of Model 4 are given in Table 3. It shows that the intersection significantly positively correlates with fundraising ability. The result indicates that the project type has a moderating effect between social recommendation and fundraising ability. The promoting effect of social recommendations on fundraising is enhanced in the medical aid crowdfunding project. Hypothesis H3 is supported. According to the study of Dawson (2014), the moderating effect of project type is given in Figure 1. Under the medical aid crowdfunding project, the social recommendation has enhanced the positive impact on project fundraising ability.

### Robustness test

This paper uses Funding Level as explained variables to verify the robustness of regression results. We further test the influence of social capital and social recommendation on the project’s fundraising ability. The regression result is given in Table 4. It shows that the positive correlation between external social capital, social recommendation, and the proportion of funds raised is still significant. The moderating effect of project type also still existed. The only difference is the regression results of *Intcapital II*. *Intcapital II* has a negative coefficient with *Funding Level*. This presents that the number of backers brought by sponsors can increase the donation amount, but it cannot promote the completion of crowdfunding goals. External social capital and the first index of internal social capital can significantly improve the completion degree of project fundraising. Social capital and social recommendation still play an important role in the proportion of funds raised. The robustness test is almost entirely passed.

**TABLE 4:** Robustness test.

	(1)	(2)	(3)	(4)
	Funding level	Funding level	Funding level	Funding level
*Goal*	−0.602*** (−33.08)		−0.643*** (−35.39)	−0.639*** (−35.07)
*Dur*	0.000 (0.22)		−0.004** (−3.03)	−0.004** (−2.94)
*Year*	0.0785* (2.14)		0.057 (1.59)	0.056 (1.58)
*Type*	−0.062 (−1.22)		0.196*** (4.01)	0.157** (3.06)
*Fans*	0.035*** (3.73)		−0.033* (−2.54)	−0.032* (−2.43)
*Prefunding*	−0.084** (−3.06)		−0.101** (−3.12)	−0.110*** (−3.38)
*Extcapital*		−0.146*** (−10.12)	0.073*** (4.87)	0.076*** (5.05)
*Intcapital I*		0.079*** (10.91)	0.023** (3.10)	0.022** (3.00)
*Intcapital II*		0.030 (1.44)	−0.044* (−2.22)	−0.044* (−2.26)
*Support*		0.463*** (24.21)	0.481*** (28.26)	0.483*** (28.37)
Support*type				0.106* (2.45)
*Constant*	7.646*** (41.34)	0.596*** (6.38)	6.423*** (35.01)	6.392*** (34.77)
Adj *R*^2^	0.267	0.157	0.373	0.374

*, **, ***Denote statistical significance at the 10, 5, and 1% levels, respectively. *T*-value is reported in parentheses.

## Discussion

### Summary of main findings

Social interaction signals such as social capital, reciprocal behavior, recommendation, and sharing in charity crowdfunding campaigns affect the performance of crowdfunding projects (Skirnevskiy et al., 2017). We study the impact of project sponsors’ social capital and social recommendations on the project’s fundraising ability. The data of this research is from 4,780 crowdfunding projects on Charity Crowdfunding of Sina MicroBlog. The conclusions are as follows: First, both external social and internal social capital significantly impact the fundraising ability of charity crowdfunding projects. An effective social network can help project sponsors attract potential supporters on the platform and expand fundraising channels for crowdfunding projects. In particular, social friends of project sponsors outside the platform can promote the acquisition of crowdfunding project resources. External social capital plays a significant role in project fundraising (Borst et al., 2018); Historical information about the sponsor’s involvement in crowdfunding projects can improve the trustworthiness of a crowdfunding campaign. Internal social capital also can significantly enhance the number of funds raised (Yin et al., 2019). Experienced project sponsors are more likely to be supported by potential backers.

Second, the communication mechanism of social recommendation can significantly promote the project’s fundraising ability. Potential backers can read and comment about the project during the crowdfunding campaign. Potential backers’ recommendations and forwarding can make the project spread more widely. Social recommendations also increase the opportunity to get project resources. The project is more recommended, the more chance it will be supported by potential backers (Vismara, 2018). The recommendation of a project becomes an important signal of project quality, which enhances the trust of potential backers in the project. Consequently, it improves the project obtaining funding (Schafer et al., 2018). Project forwarding, liking, and other communications are necessary for the charity crowdfunding campaign. Social recommendation is an effective communication mechanism (Yin et al., 2019). Social interaction improves the enthusiasm of potential backers to support the project (Kim et al., 2017).

Third, the social recommendation can effectively improve the fundraising ability in medical aid crowdfunding projects. The promotion effect of social recommendation on project fundraising performance is amplified in the medical aid crowdfunding project. Investment decisions of potential backers are context-dependence. Medical aid is related to people’s lives and health. This kind of demand is more likely to touch donors’ emotions. A medical aid crowdfunding project is easier to accept by the public. This conclusion also proves the context-dependence of individual decision-making, revealing that prosocial behavior plays a crucial role in charity crowdfunding (Hong et al., 2018).

### Implications for research

First, this study introduces the external and internal social capital to explore the charity crowdfunding influencing factors. We expand the influencing factors of charity crowdfunding performance from the perspective of social capital. Our findings enrich relevant research. The current research on crowdfunding mainly focuses on reward crowdfunding and neglects the influencing factors of charity crowdfunding from the perspective of social interaction.

Zheng et al. (2014) is one of the first to examine the effects of the three dimensions of social capital on crowdfunding performance. Their research has inspired the follow-up research, which offers a good lens for understanding crowdfunding. However, there are still some shortcomings that could be improved. For example, there are overlaps between different dimensions of social capital. The boundaries of different dimensions of social capital are ambiguous (Madrazo-Lemarroy et al., 2019). Thus, a precise classification of social capital is needed better to understand crowdfunding (Cai et al., 2021).

The studies of Colombo et al. (2015) and Skirnevskiy et al. (2017) focused on social capital within the platform and paid relatively little attention to social capital outside the platform. Moreover, Mollick (2014), Zheng et al. (2014), Colombo et al. (2015) and Skirnevskiy et al. (2017), and other studies are based on reward crowdfunding projects, while this study is based on charity crowdfunding projects. Our research divides social capital into external and internal dimensions, which can help us better investigate social capital’s promotion effect on charity crowdfunding projects. Our analysis also extends the social capital theory application field (Mollick, 2014; Zheng et al., 2014; Colombo et al., 2015; Skirnevskiy et al., 2017).

Second, this study expands previous research on charity crowdfunding in terms of social recommendations. We discuss the influencing factors of charity crowdfunding performance from the perspective of social recommendation (Sura et al., 2017; Li et al., 2018; Peng et al., 2022). Research on traditional charity crowdfunding is mainly based on platform characteristics, fundraising goals, project description, narrative style, and other information. Existing literature rarely include social interaction information between backers and projects. Whether structural social capital or relational social capital, previous studies based on social capital theory are not directly related to the current project. Moreover, the factors are relatively static external information and do not include the current crowdfunding project’s dynamic process of social activities (Skirnevskiy et al., 2017).

Dynamic signals such as Likes and forwards received by the project indicate the support level of the project and the sponsors’ trustworthiness. It is also an important factor affecting the fundraising ability of the project (Wang et al., 2018). When investigating the influencing factors of crowdfunding performance, this study considers recommended information for the project.

Third, Our study obtains the microscopic behavior data of project on crowdfunding platform through the web crawler program. Then, we explore influencing factors of fundraising ability in a charity crowdfunding project based on actual objective data. We provide objective data for understanding the operation process of charity crowdfunding. Based on the operation data of crowdfunding platforms, this paper analyses the influencing factors of charity crowdfunding in China. The conclusion may be inconsistent with the existing law because there is a deviation between individual subjective judgment and actual behavior. It is also a significant limitation of traditional questionnaire research. Sura et al. (2017), Li et al. (2018), and Bagheri et al. (2019) mainly employ questionnaires to discuss the impact of project fundraising performance which are not real projects on crowdfunding platforms.

### Implication for practice

First, social media users are the foundation for projects to raise funds. Social media users are not only potential backers but also an important symbol of project trustworthiness. Their social interaction can also transmit valuable signals and attract more potential backers. For project sponsors, they need to pay attention to the value of their social friends. On the one hand, project sponsors can take advantage of their resources to get as many social friends as possible, which will increase the number of potential backers and project advocates. On the other hand, project sponsors need to use their social friends well. For example, project sponsors adopt a specific incentive mechanism to encourage social friends to attract more social media users. Social media users can become new social friends who can pay attention to charity crowdfunding projects.

Second, internal social capital is also a remarkable factor in improving the fundraising ability of projects. The interaction between sponsors and potential backers can improve potential backers’ comprehensive understanding of the project. Project sponsors need to focus on cultivating the quality of internal social capital and the strength of relationships with social friends. For example, project sponsors can conduct propaganda and promote their crowdfunding projects through platforms such as Weibo, which will improve the quality of the relationship with potential backers.

Third, the social recommendation of the project can significantly promote the project to access funds. The project sponsors need to get more support from social friends. In this case, project sponsors can set up some incentive mechanisms to get social recommendations. For example, with the help of live broadcasting, reward-forwarding behavior, or building communication groups, the project can get as much recognition from social media users as possible, spreading the project effectively. To gain the trust of more potential backers, project sponsors must also pay attention to building relationships and burnishing their reputations.

Fourth, considering the efficiency and convenience of network information dissemination, social norms derived from network are more likely to affect more people. In network, people’s decision-making behaviors are recorded, and some information forms social capital and social recommendations. These are important social norms in network which bind people’s behaviors. As concluded in this study, social capital and social recommendations have a significant impact on crowdfunding performance, social norms in network motive people’s positive behaviors and contribute to social harmony and stability. Therefore, we need to pay attention to how to cultivate social norms in network and take full advantages it.

### Limitations and future research

There are many factors influencing project crowdfunding performance. This study mainly focuses on the crowdfunding projects on Charity Crowdfunding of Sina MicroBlog. Future research can select crowdfunding projects on other social platforms to further verify the effect of social capital, social recommendation, and other factors on fundraising ability. The measurement of social capital is of great importance to this study. However, there may be limitations in measuring social capital caused by data acquisition limitations. To better investigate the impact of social capital on fundraising ability, various data mining methods need to be fully utilized in the following research.

## Conclusion

Current studies mainly focus on reward crowdfunding projects. The impact of social networks on the fundraising ability of charity crowdfunding projects is rarely discussed using crowdfunding platforms’ data. Taking the crowdfunding projects in Charity Crowdfunding of Sina MicroBlog in China as a sample, this paper analyses how social capital and social recommendation influence the performance of crowdfunding projects. This study contributes to the research on the influencing factors of the fundraising ability of charity crowdfunding.

## Data availability statement

The datasets presented in this study can be found in online repositories. The names of the repository/repositories and accession number(s) can be found in the article/supplementary material.

## Author contributions

CL and JW conceived the idea of the manuscript, designed the research, collected, analyzed the data, and wrote the manuscript. MP, XL, and WG modified the manuscript. All authors have read and approved the final manuscript.

## References

[B1] Bagheri A. Chitsazan H. Ebrahimi A. (2019). Crowdfunding motivations: A focus on donors’ perspectives. *Technol. Forecast. Soc. Change* 146 218–232. 10.1016/j.techfore.2019.05.002

[B2] Banerjee A. V. (1992). A simple model of herd behavior. *Q. J. Econ.* 107 797–817. 10.2307/2118364

[B3] Borst I. Moser C. Ferguson J. (2018). From friend funding to crowdfunding: Relevance of relationships, social media, and platform activities to crowdfunding performance. *New Media Soc.* 20 1396–1414. 10.1177/1461444817694599 30581357 PMC6256715

[B4] Butticè V. Colombo M. G. Wright M. (2017). Serial Crowdfunding, Social Capital, and Project Success. *Entrep. Theory Practice* 41 183–207. 10.1111/etap.12271

[B5] Cai W. X. Polzin F. And Stam E. (2021). Crowdfunding and social capital: A systematic review using a dynamic perspective. *Technol. Forecast. Soc. Change* 162:120412. 10.1016/j.techfore.2020.120412

[B6] Calic G. Mosakowski E. (2016). Kicking off social entrepreneurship: How a sustainability orientation influences crowdfunding success. *J. Manag. Stud.* 53 738–767. 10.1111/joms.12201

[B7] Colombo M. G. Franzoni C. Rossi-Lamastra C. (2015). Internal Social Capital and the Attraction of Early Contributions in Crowdfunding. *Entrep. Theory Practice* 39 75–100. 10.1111/etap.12118

[B8] Davies E. Giovannetti E. (2018). Signalling experience and reciprocity to temper asymmetric information in crowdfunding evidence from 10,000 projects. *Technol. Forecast. Soc. Change* 133 118–131. 10.1016/j.techfore.2018.03.011

[B9] Dawson J. F. (2014). Moderation in management research: What, why, when, and how. *J. Bus. Psychol.* 29 1–19. 10.1007/s10869-013-9308-7

[B10] Donovan J. (2021). Financial Reporting and Entrepreneurial Finance: Evidence from Equity Crowdfunding. *Manag. Sci.* 67 7214–7237. 10.1287/mnsc.2020.3810 19642375

[B11] Hervé F. Manthé E. Sannajust A. Schwienbacher A. (2019). Determinants of individual investment decisions in investment-based crowdfunding. *J. Bus. Finance Account.* 46 762–783. 10.1111/jbfa.12372

[B12] Hildebrand T. Puri M. Rocholl J. (2017). Adverse incentives in crowdfunding. *Manag. Sci.* 63 587–608. 10.1287/mnsc.2015.2339 19642375

[B13] Hong Y. Hu Y. Burtch G. (2018). Embeddedness, Pro-Sociality, and Social Influence: Evidence from Online Crowdfunding. *MIS Q.* 42 1211–1224. 10.25300/MISQ/2018/14105

[B14] Khadjavi M. (2017). Indirect reciprocity and charitable giving—evidence from a field experiment. *Manag. Sci.* 63 3708–3717. 10.1287/mnsc.2016.2519 19642375

[B15] Kim T. Por M. H. Yang S.-B. (2017). Winning the crowd in online fundraising platforms: The roles of founder and project features. *Electron. Commer. Res. Appl.* 25 86–94. 10.1016/j.elerap.2017.09.002

[B16] Kromidha E. Robson P. (2016). Social identity and signalling success factors in online crowdfunding. *Entrep. Reg. Dev.* 28 605–629. 10.1080/08985626.2016.1198425

[B17] Kuppuswamy V. Bayus B. L. (2017). Does my contribution to your crowdfunding project matter? *J. Bus. Venture.* 32 72–89. 10.1016/j.jbusvent.2016.10.004

[B18] Li J. Zhang Y. Niu X. (2021). The COVID-19 pandemic reduces trust behavior. *Econ. Lett.* 199:109700. 10.1016/j.econlet.2020.109700 PMC9754319 36540697

[B19] Li Y. Z. He T. L. Song Y. R. Yang Z. Zhou R. T. (2018). Factors impacting donors’ intention to donate to charitable crowd-funding projects in China: A UTAUT-based model. *Inf. Commun. Soc.* 21 404–415. 10.1080/1369118x.2017.1282530

[B20] Lin M. Prabhala N. R. Viswanathan S. (2013). Judging Borrowers by the Company They Keep: Friendship Networks and Information Asymmetry in Online Peer-to-Peer Lending. *Manag. Sci.* 59 17–35. 10.1287/mnsc.1120.1560 19642375

[B21] Lins K. V. Servaes H. Tamayo A. (2017). Social capital, trust, and firm performance: The value of corporate social responsibility during the financial crisis. *J. Finance* 72 1785–1824. 10.1111/jofi.12505

[B22] Liu L. Suh A. Wagner C. (2018). Empathy or perceived credibility? An empirical study on individual donation behavior in charitable crowdfunding. *Internet Res.* 28 623–651. 10.1108/intr-06-2017-0240

[B23] Madrazo-Lemarroy P. Barajas-Portas K. Tovar M. E. L. (2019). Analyzing campaign’s outcome in reward-based crowdfunding Social capital as a determinant factor. *Internet Res.* 29 1171–1189. 10.1108/intr-03-2018-0115

[B24] Mollick E. (2014). The dynamics of crowdfunding: An exploratory study. *J. Bus. Venture.* 29 1–16. 10.1016/j.jbusvent.2013.06.005

[B25] Peng Y. Li Y. Wei L. (2022). Positive Sentiment and the Donation Amount: Social Norms in Crowdfunding Donations During the COVID-19 Pandemic. *Front. Psychol.* 13 818510–818510. 10.3389/fpsyg.2022.818510 35265015 PMC8901185

[B26] Proelss J. Schweizer D. Zhou T. (2020). Economics of philanthropy—evidence from health crowdfunding. *Small Bus. Econ.* 57 999–1026. 10.1007/s11187-020-00336-w

[B27] Schafer M. S. Metag J. Feustle J. Herzog L. (2018). Selling science 2.0: What scientific projects receive crowdfunding online? *Public Underst. Sci.* 27 496–514. 10.1177/0963662516668771 27647666 PMC6041758

[B28] Skirnevskiy V. Bendig D. Brettel M. (2017). The Influence of Internal Social Capital on Serial Creators’ Success in Crowdfunding. *Entrep. Theory Practice* 41 209–236. 10.1111/etap.12272

[B29] Sura S. Ahn J. Lee O. (2017). Factors influencing intention to donate via social network site (SNS): From Asian’s perspective. *Telemat. Inform.* 34 164–176. 10.1016/j.tele.2016.04.007

[B30] Vismara S. (2018). Information Cascades Among Investors in Equity Crowdfunding. *Entrep. Theory Practice* 42 467–497. 10.1111/etap.12261

[B31] Wang N. Li Q. Liang H. Ye T. Ge S. (2018). Understanding the importance of interaction between creators and backers in crowdfunding success. *Electron. Commer. Res. Appl.* 27 106–117. 10.1016/j.elerap.2017.12.004

[B32] Yin C. Liu L. Mirkovski K. (2019). Does more crowd participation bring more value to crowdfunding projects? The perspective of crowd capital. *Internet Res.* 29 1149–1170. 10.1108/intr-03-2018-0103

[B33] Zhang H. Chen W. (2019). Backer Motivation in Crowdfunding New Product Ideas: Is It about You or Is It about Me? *J. Prod. Innov. Manag.* 36 241–262. 10.1111/jpim.12477

[B34] Zheng H. Li D. Jing W. Yun X. (2014). The role of multidimensional social capital in crowdfunding: A comparative study in China and US. *Inf. Manag.* 51 488–496. 10.1016/j.im.2014.03.003

